# Supplementary material for: Effect of health education intervention on treatment adherence and health status in patients with Chronic obstruction pulmonary disease: A random control trial
Source: PLoS One. 2025 Jun 24;20(6):e0325192. doi: 10.1371/journal.pone.0325192 (PMC12186925; doi:10.1371/journal.pone.0325192)
Supplement: S2 File — (PDF) [file pone.0325192.s002.pdf]

STUDY PROTOCOL

1. PARTICIPANTS AND SITES

1.1. Participants

** Inclusion criteria*

- Patients were diagnosed with COPD according to GOLD 2018 criteria;
- Received stable home treatment with inhaled medications;
- No acute episodes, including acute episodes due to chronic diseases requiring hospitalization for at least 3 months;
- Able to speak, read and understand Vietnamese;
- Participants had and knew how to use a smartphone with an Internet connection;
- Voluntarily participated in the study.

**Exclusion criteria*

- History of bronchial asthma, allergic rhinitis, lung surgery, or respiratory diseases.
- People with mental disorders or other serious illnesses.

1.2. Research site: Examination Department of Da Nang C Hospital as the intervention site and Examination Department of Da Nang Hospital for Lung Disease as the control site.

2. RESEARCH TIME

- The pre-intervention data collection period spans from April 2021 to October 2021.
- Content development for the intervention occurs from November 2021 to April 2022.
- The intervention itself takes place from April 2022 to June 2022, lasting three months.
- Post-intervention assessments are conducted from July 2022 to August 2022.

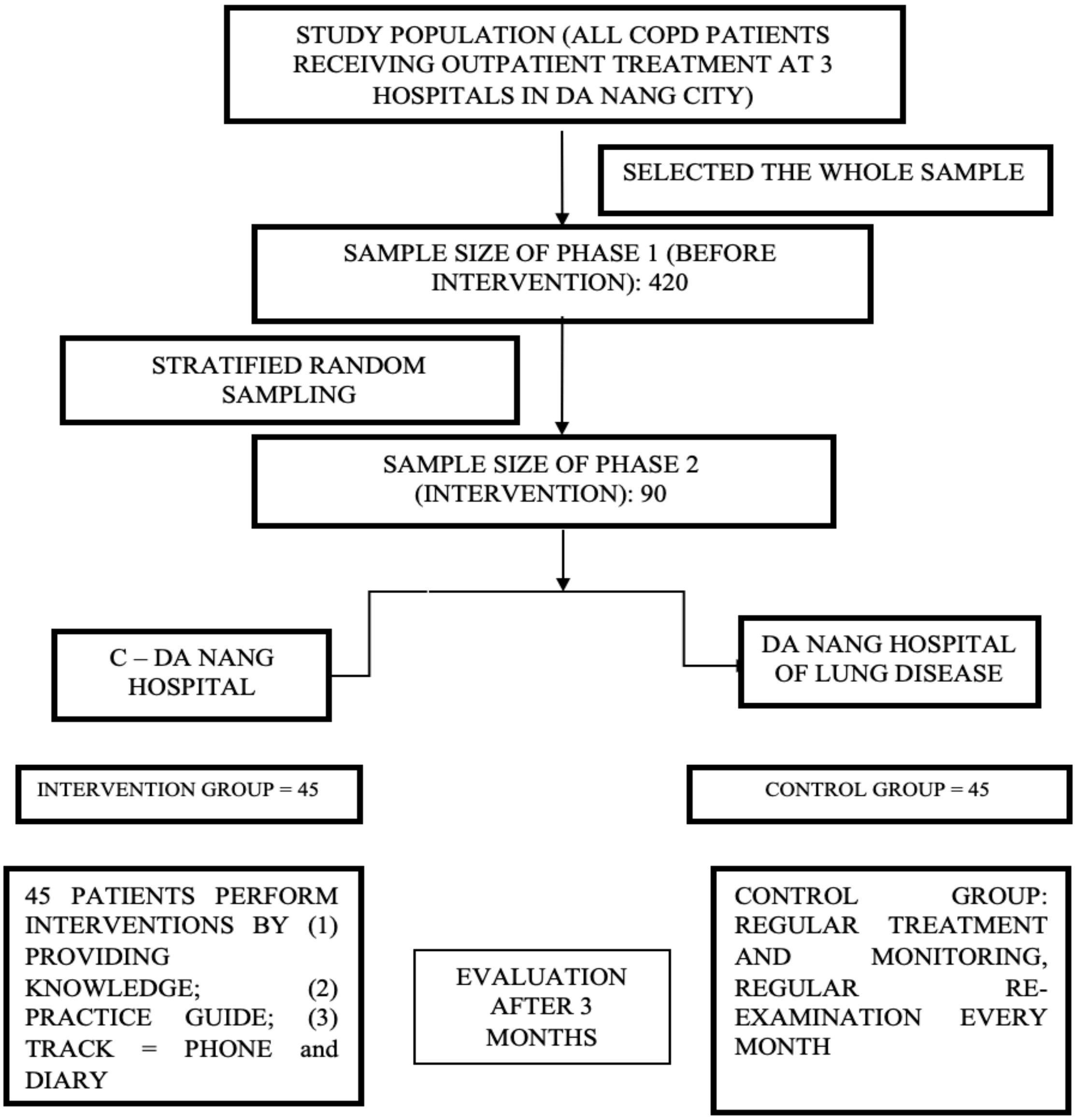

Figure 1. Study flowchart

3. INTERVENTION PROCESS

We used a randomized controlled intervention study design

The two main research phases include:

- Phase 1: Pre-intervention assessment, cross-sectional description of treatment adherence and related factors of people with COPD who are receiving outpatient treatment at medical facilities in Da Nang City, specifically at the Outpatient Department of three hospitals: Da Nang C Hospital, Da Nang Hospital, and Da Nang Hospital for Lung Disease and related factors.

- Phase 2: Evaluation of intervention results after 3 months with the following steps:

+ Step 1: Develop intervention content, and verify the acceptability, appropriateness, and feasibility of the intervention program through consulting experts and patients (for specific details, see the section "List of published works of the author related to the thesis" in order number 4 and 5).

+ Step 2: Implement the intervention.

+ Step 3: Post-intervention assessment is performed comparing before and after intervention and comparing between the intervention group and control group (see Figure 1)

4. DEVELOP AN INTERVENTION PROGRAM

4.1. Criteria and principles for developing intervention content

To ensure the effectiveness of intervention activities, the intervention model is built based on 3 criteria: (1) appropriateness and feasibility; (2) sustainability; and (3) applicability.

- Principles to ensure appropriateness and feasibility:

+ Based on the existing system;

+ In line with the national program for the prevention of COPD;

+ Acceptable funding;

+ Intervention content is easy to apply.

- Principles to ensure sustainability:

+ In line with practical conditions, hospitals all have plans to build a model of periodic health education for patients, especially those with COPD;

+ In line with the psychological characteristics of patients, meeting the needs/deficiencies and desires of patients and their families.

- Principles to ensure applicability:

+ Intervention content is presented clearly/briefly, with easy-to-understand words, convenient and easy to implement;

+ Compact/beautiful/attractive form to participants.

4.2. Intervention program

Based on a review of various documents and the analysis of numerous studies from around the world and Vietnam, combined with Ajzen's Planned Behavior Theory model (1991), we identified several barriers that lead to non-compliance with treatment. These barriers include a lack of awareness about the disease, a lack of

confidence in the effectiveness of treatment medications, and insufficient understanding of the benefits of breathing exercises. Many patients maintain a daily routine of breathing exercises but still hold incorrect attitudes toward treatment, resulting in poor adherence. Additionally, some patients may be aware of their condition and trust the prescribed medications; however, they may still rely too heavily on their personal experiences, leading to a subjective approach that hinders their adherence to the recommended treatment protocols.

Table 1. Intervention program based on the theory of planned behavior

Factors	Contents	Activities	Evaluate
- Attitude toward behavior;<br>-Subjective norms;<br>-Perceived behavioral control.	- Assess patient knowledge.<br>- Assess practice in using inhaled medications.<br>- Assess practice in breathing exercises.	- Provide consultation to improve patient knowledge.<br>- Provide training on inhaler use techniques and practice breathing exercises at the hospital for patients and their families.<br>- Apply periodic monitoring methods via phone to remind patients of treatment compliance, provide documents, instructional videos on inhaler use and breathing exercises for patients and their families to view at any time.<br>- Provide instructions on keeping a diary.	- COPD- Q;<br>- Inhaled medication practice checklist;<br>- Breathing practice checklist;<br>- Medication adherence;<br>- mMRC and CAT scores<br>- Lung function measurement;<br>- Percentage of participants in zalo group calls;<br>- Diary.

Table 2. Summary of solutions and interventions

No.	Intervention components	Planned activities
1	Objectives	(1) improve patient knowledge about COPD, (2) promote COPD self-management behavior, and (3) practice correct inhaler technique
2	Participants	Outpatients diagnosed with COPD who are taking home bronchodilators with oral or inhaled medications
3	Number of interventions	3 sessions, each session 1 week apart (**in hospital**)<br>10 a group phone call via the Zalo chat group within 10 weeks (**at home**)
4	Time for each intervention session	30 - 45 min (**in hospital**)<br>3 – 5 min, no more than 10 min (**at home**)
5	Type of intervention	**In hospital**: large group intervention for sessions 1 and 2; and intervention in small groups of 4-5 people/group, or pairing in groups in session 3<br>**At home**: once a week in the 8-9 am time frame on Wednesday of the week, the researcher will make a group phone call via the Zalo chat group
6	Contents of intervention	**In hospital**, include 3 parts:<br>Part 1: Focus on improving the patient's knowledge.<br>Part 2: Focused on guiding self-management measures.<br>Part 3: Indicates common errors and solutions when using inhaled drugs; Practical Guide to Inhaled Drugs Inhaled PMDI metered dose inhaler, Turbuhaler. Instruct the patient to create a paper-based action plan based on the appropriate goals for the patient.<br>**At home:** patients self-reported drug use; drug side effects (if any); number of episodes of dyspnea per week, amount, and color of sputum; and discuss with researcher about self-care skills, breathing exercises; write diary.

No.	Intervention components	Planned activities
7	Implementation	Direct implementation at the Outpatient clinic<br>Online calling at home
8	Methods	Presentation, group discussion, face-to-face practice, online calling
9	Post-intervention evaluation time	After 1 and 3 months
10	Post-intervention measurement tool	COPD – Q, IUS-V, checklist, TAI
11	Hand-outs	The handbook has been compiled based on documents of GOLD 2020 and documents of the national target program for prevention of chronic obstructive pulmonary disease and asthma.

4.3. Implementing organization

The intervention activities were carried out at the Outpatient Department of Da Nang C Hospital. The subjects participating in the intervention included: PhD students; 02 respiratory nurses; and 02 lecturers from Da Nang University of Medical Technology and Pharmacy.

4.3.1. Intervention preparation process

- Organize group discussions (with patients and medical staff of the department) to detect barriers to treatment compliance. At the same time, consult with experts to determine the content and method of intervention.
- Agree on the content and form of intervention.
- Develop a detailed intervention plan within 3 months.
- Closely coordinate with the examination department to organize the implementation of the intervention.
- Monitor the implementation of the intervention, including Intervention content, implementers, and number of participants according to the pre-designed form.

To ensure the effectiveness and sustainability of the intervention, the intervention content is implemented according to the principles: (i) simple, easy to understand; (ii) suitable for the psychological characteristics of the subject; and (iii) suitable for practice (only providing what the patient lacks).

4.3.2. Implement intervention activities for the intervention group

***Session 1 (week 1):** Focus on improving patient knowledge (large group of 45 people directly in the hospital hall).

Table 3. Intervention implementation process week 1

No	Contents	Required	Note
1	Greet in a friendly manner.	Ensure good communication skills.	
2	Ask and listen to the participants talk about COPD and how to self-manage the disease at home.	Introduce the purpose of the consultation.	Be careful to ask slowly, loudly and clearly.
3	Identify the knowledge and skills that the participants lack in self-management at home.	Ask patients about the content that needs to be communicated about COPD.	
4	Show the ppt to show the knowledge that the participants lack.	Pay attention and check the content that patients have not listed.	
5	Explain the benefits of adhering to inhaled medication even when there are no symptoms of the disease as well as the benefits of practicing breathing exercises.	Use communication materials to supplement the knowledge, skills, and self-management skills that patients lack.	Be careful to ask the audience if they can hear clearly?
6	Encourage the participants to practice self-management at home with specific activities.	Ask them to speak slowly, loudly, and clearly.	
7	Guide the participants to use the handout.	For example: Quit smoking, comply with taking medication at home, comply with practicing breathing exercises at home, etc.	
8	Ask the participants to	Distribute the prepared	

No	Contents	Required	Note
	repeat the knowledge and practice of self-management at home.	materials to patients and ask if they can read them. Instruct patients to focus on	
9	Agree with the participants on what they must do to prevent acute attacks of the disease at home.	the content that needs attention.	
10	Take time to answer questions and evaluate the session.		
11	Greet and close.		

***Session 2 (week 2):** Focus on improving self-care practice skills (divided into 7 small groups of 5-7 people/group), directly at the hospital hall)

Table 4. Intervention implementation process week 2

No	Contents	Required	Note
1	Warm greetings.	Ensure good communication skills. Introduce the purpose of the consultation.	
2	Review the content of session 1 through game questions.	Ask the participants about the content that needs to be communicated about the COPD provided in session 1.	Remember to ask slowly, loudly and clearly.
3		Pay attention and check the steps that the participants did not perform or made mistakes.	
4	Divide into small groups (5-7 people/group) to demonstrate the technique of using inhaled drugs and the technique of practicing breathing exercises in the group.	Show the video on how to use inhalers and practice breathing exercises.	Slow down each step, emphasizing common errors and how to fix them

No	Contents	Required	Note
5	Practice the sample technique of using inhaled drugs (simulating the model) and practice breathing exercises.	Ask them to speak slowly, loudly, and clearly.	Be careful to ask the patient if he/she can hear clearly?
6	The participants practice again.	Provide the link.	Be careful to observe and tick the steps that the patient cannot perform or has errors. Point out the remaining errors and practice again until it is achieved.
7	Instruct the participants on how to download videos on how to use inhaled drugs and practice breathing exercises on smartphones to watch at any time.	Show the participants the steps to use inhalers and practice breathing exercises in the handout and ask if they can read it.	
8	Instruct the participants on how to use the handout.	Only ask the participants to repeat the content that the researcher has just provided to the participants.	
9	Ask the participants to review the knowledge and skills of practicing self-management of the disease at home after 2 sessions.	Repeat the content that the participants do not remember.	
10	Agree with the participants on what they must do to prevent acute attacks of the disease at home.		
11	Take time to answer questions and evaluate the session.	Instructions patients for using Zalo for group calls.	Ensure 100% of the patients participate in zalo groups.
12	Create a zalo group of 5-7	Distribute diary and writing	Ensure 100% of the

No	Contents	Required	Note
	people in the group with the researcher.	instructions.	patients understand and know how to write a diary.
13	Instructions for writing a diary.		

***From the 3rd session to the 12th session**: Implement a method of periodic weekly monitoring via phone at home: Periodically once a week between 8-9 am (pre-arranged) on Wednesday of the week (for 10 consecutive weeks), the researcher will make a group phone call via the Zalo chat group, the time for each phone call is 10-15 minutes and no more than 30 minutes for 1 call. The researcher is advised to turn on their mobile phones in the morning; for the researcher who does not participate in the group call, the collaborator will call via their phone number to remind them to join the Zalo chat group, the private call will be made 3 times, each time 5 minutes apart, ensuring that the group call has enough participants. Participants were instructed not to tell or share the phone call with others, if the participant lost or changed his/her phone number, a new phone number would be provided immediately after he/she had informed the research team. The content of the calls included: Reminding the patient about treatment compliance, the need to comply with treatment; reminding the patient to record the side effects of the medication if any; reminding the patient to record the number of dyspnea episodes/week, the amount of sputum, the color of sputum; difficulties or errors that may occur when practicing using inhaled medication and practicing breathing exercises.

***At the end of 10 weeks** of home monitoring via phone calls, perform post-intervention assessment.

Intervention tools:

- Power point lectures, illustrations;
- Sample drug delivery tools: MDI metered dose inhaler, Accuhaler dry powder inhaler, Turbuhaler;
- Patient simulation model;
- Phone with zalo application function;
- Prepare tools for consultation session: Projector, computer, leaflets...;

- Intervention method: Theoretical teaching and practical demonstration;

- Organization method: Presentation, group discussion, Q&A, demonstration of sample operations.

5. MEASURES TO LIMIT ERRORS

In this study, the main errors encountered were sampling errors and data collection errors. To ensure reliability and limit errors, we calculated the study sample size to be relatively large and representative of major hospitals in Da Nang city. The selection of individuals participating in the study was carried out following the correct procedure.

To overcome errors in information collection, the research team took the following measures:

- The toolkit was designed to be simple, easy to understand, and easy to answer, the contents were arranged logically and tested before use.

- Selection of investigators to measure lung function: They are people with clinical experience. Lung function measurements are performed under the supervision of head nurses specializing in respiratory medicine.

- Data collection was carried out by the graduate students themselves. Data collection was conducted strictly: (i) Graduate students reviewed and re-checked 100% of the questionnaires that had been interviewed; (ii) The researcher re-interviewed 10% of the surveyed questionnaires; Survey questionnaires that were assessed as not highly reliable for various reasons were checked and re-interviewed. If they did not meet the requirements, they were eliminated and not included in the data analysis.

6. ETHICAL CONSIDERATION

- The study was conducted in accordance with the thesis outline approved by the Outline Approval Council and the Medical Ethics Council of Nam Dinh University of Nursing, as per Decision No. 1681/GCN-HDDD dated August 2, 2021.
- Approval was obtained from Da Nang C Hospital, Da Nang Hospital, and Da Nang Hospital for Lung Disease.
- Participants were individuals with Chronic Obstructive Pulmonary Disease (COPD) who attended the outpatient clinics of the three hospitals mentioned above. They willingly agreed to participate in the study by signing a consent form that provided by the researcher at the first-time meeting.

- Since the study was conducted during the COVID-19 epidemic control period in Da Nang City, we took precautions to ensure the safety of all participants. This involved close coordination with the hospitals to screen and examine COVID-19 patients, organize patient flow, and create separate pathways for study participants.
- Participants in the intervention group were informed about the importance of confidentiality and committed to not sharing the knowledge they gained during the study with others. After completing the intervention, all subjects in the control group participated in two health counseling sessions and received documentation containing similar information to that provided to the intervention group.
